# Ambient Air Pollution and Daily Hospital Admissions for Respiratory Disease in Children in Guiyang, China

**DOI:** 10.3389/fped.2019.00400

**Published:** 2019-10-04

**Authors:** Hao Zhou, Tianqi Wang, Fang Zhou, Ye Liu, Weiqing Zhao, Xike Wang, Heng Chen, Yuxia Cui

**Affiliations:** ^1^Department of Pediatrics, Guizhou Provincial People's Hospital, Medical College of Guizhou University, Guiyang, China; ^2^Neurology Department, Children's Hospital of Fudan University, Shanghai, China; ^3^Otolaryngological Department, Guizhou Provincial People's Hospital, Medical College of Guizhou University, Guiyang, China; ^4^Medical College of Guizhou University, Guiyang, China

**Keywords:** air pollution, respiratory disease, daily hospital admissions, economic cost of hospitalization, children

## Abstract

**Objectives:** To investigate the association between ambient air pollutant exposure and daily hospital admissions for respiratory diseases in children in Guiyang.

**Methods:** Clinical data of pediatric inpatients with respiratory disease from 2009 to 2016 in Guizhou Provincial People's Hospital and PM2.5, NO_2_, PM10, and SO_2_ concentration data were retrieved. A canonical correlation analysis (CCA) was applied to analyse the association between air pollutants and daily hospital admissions for respiratory diseases. A reproducibility analysis was applied to analyse the association between air pollution and the duration and direct cost of hospitalization. The support vector regression (SVR) method was applied to determine whether air pollution data could predict the daily hospital admissions for the upcoming day.

**Results:** A total of 10,876 inpatients with respiratory diseases were included between January 1, 2009 and December 31, 2016. The CCA showed significant correlations between air pollution and daily hospital admissions (*r* = 0.3564, *p* < 0.001), the duration of hospitalization (*r* = 0.2911, *p* < 0.001) and the economic cost of hospitalization (*r* = 0.2933, *p* < 0.001) for respiratory disease. PM10 contributed most to daily hospital admissions for respiratory disease; the concentration the day before hospitalization contributed most to the daily hospital admissions for respiratory disease. There was a slightly stronger correlation between air pollution and respiratory disease in children aged 2–18 years (*R* = 0.36 vs. *R* = 0.31 in those under 2 years old). No significant difference was found between male and female patients. The prediction analysis showed that air pollution could successfully predict daily pediatric inpatient hospital admissions (*R* = 0.378, permutation *p* < 0.001).

**Conclusions:** Air pollution was significantly associated with hospital admissions, hospitalization duration and the economic cost of hospitalization in children with respiratory diseases. The maximum effect occurred on the day before hospitalization. The effect of PM10 on daily pediatric inpatient hospital admissions for respiratory disease was the greatest among the pollutants evaluated.

## Introduction

Air pollution is an important environmental risk factor for human health (1). Evidence is mounting that ambient air pollution exposure is significantly associated with respiratory diseases (2, 3). Ambient air pollution, such as nitrogen dioxide (NO_2_), sulfur dioxide (SO_2_), and particulate matter (PM), are associated with mortality and morbidity induced by respiratory diseases (4–6). The relationship between air pollutants and respiratory hospital admissions has been reported both in developed countries and in developing countries (7, 8). Other studies have shown an adverse effect of ambient air pollution exposure on morbidity and mortality, as well as on healthcare costs (9–12).

Children are particularly susceptible to ambient air pollution due to their physiological and behavioral characteristics. A previous study found that children were the most susceptible to hospital admission for respiratory disease associated with PM levels and meteorological factors (13). China is experiencing severe air pollution and related health effects due to rapid economic and social development (14). According to the World Health Organization air pollution database (15), the annual population weighted PM10 concentration of 90 μg/m^3^ in China was much higher than that in the United States (21 μg/m^3^) (15). In China, PM2.5 caused approximately 17.2–57.0 billion Yuan in the total medical cost of outpatient care for respiratory diseases in 2014, accounting for 0.5–1.6% of the total national health expenditure (16).

However, previous studies on air pollution and respiratory diseases were mainly concentrated in western countries and northern industrial cities in China, such as Beijing, Jinan, and Lanzhou (17–19), and the pollution levels in other cities has received limited attention, especially in less industrial cities the southwest. In addition, few studies have estimated the association between individual respiratory disease hospitalization costs and ambient air pollution in children (20). The large pediatric population and high air pollution level in China provide potential for assessing the impact of air pollution on respiratory disease cost. The present study aimed to investigate the association between ambient air pollutant exposure (PM2.5, NO_2_, PM10, and SO_2_) and daily hospital admissions and economic cost for respiratory diseases in children in Guiyang city, Southwest China. The association between the duration of hospitalization and ambient air pollutant exposure was also analyzed.

## Materials and Methods

### Study Site

The present study was conducted in Guizhou Provincial People's Hospital, Guiyang, Guizhou Province, Southwest China, which is located between 106°07′ and 107°17′ East longitude and between 26°11′ and 27°22′ North latitude. There are more than 4.6 million inhabitants in Guiyang. Guiyang is a relatively poor and economically undeveloped city in China. Guizhou Provincial People's Hospital is a 3,000-bed tertiary teaching hospital located in Guizhou province. In 2018, 95,500 patients were discharged after an average hospital stay of 9.27 days. The air condition data between 2009 and 2016 were well-documented in the data center of the Environmental Protection Bureau in Guiyang city. There were eight monitoring stations (seven urban sites and one rural site) located in Guiyang city during the period of 2002–2009, and there nine monitoring stations (eight urban sites and one rural site) during the 2010–2012 period. There were 10 monitoring stations (eight urban sites and two rural sites) from 2013 to 2016. Data on the daily mean levels of air pollutants were obtained from the fixed monitoring stations. The measurements at the monitoring stations were taken continuously every day.

### Clinical and Atmospheric Information

The clinical data of pediatric inpatients with respiratory disease who were hospitalized in Guizhou Provincial People's Hospital from January 1, 2009, to December 31, 2016, were extracted from the Health Information System (HIS). The data of pediatric inpatients aged below 18 years and diagnosed with respiratory diseases with International Classification of Diseases 10th revision (ICD-10) code J00-J98 were collected. The extracted data included age, gender, admission date, discharge date, diagnosis based on ICD-10 codes, and the economic cost of the hospitalization. The study was approved by the institutional review board of Guizhou Provincial People's Hospital.

The PM2.5, NO_2_, PM10, and SO_2_ concentration data were retrieved from the data center of the Environmental Protection Bureau in Guiyang city. We used the data of the monitoring stations in Guiyang city to match the medical data. PM10 referred to particles with an aerodynamic diameter ≤10 μm and PM2.5 referred to those with a diameter ≤2.5 μm.

### Analytical Procedures

#### Relationship Between Air Pollution and Daily Hospital Admissions

In the present study, the air pollution status was measured by the daily mean concentration of SO_2_ (μg/m^3^), NO_2_ (μg/m^3^), PM10 (μg/m^3^), and PM2.5 (μg/m^3^) at each fixed monitoring station. To explore the relationship of air pollution with daily hospital admissions (inpatient amount) and economic cost, we performed a canonical correlation analysis (CCA), as the air pollution was measured by multiple indices. Previous studies have shown that there is a lag correlation between air pollution and respiratory disease (20). We included the recent 1-week air pollution measures in the CCA model. All measures were standardized by a z-transformation.

#### Canonical Correlation

Canonical correlation analysis is a way of inferring information from cross-covariance matrices (21). For example, there are two column vectors$,\;X={\left(x_{1},\ldots ,x_{n}\right)}^{{}^{\prime }}\;$and $Y={\left(y_{1},\ldots ,y_{m}\right)}^{{}^{\prime }}$. The CCA will find linear combinations of X and Y that have maximum correlation with each other. In mathematics, CCA seeks vectors *a* and *b* to maximize the correlation between *a*^*T*^*X* and *b*^*T*^*Y*. In the present study, there are multiple air pollution measures and a single health cost measure. The CCA seeks a weight vector, w, to maximize the correlation between $w^{T}V_{air\;}$and *V*_*inpatient*_. A Rao approximate F statistic, which is embedded in MATLAB, was applied to determine the significance level of the correlation. The value of w indicated the size of the effect of a single air pollution measure on health cost.

#### Reproducibility Analysis

The inpatient cost only accounted for the number of people who contracted respiratory disease but ignored the severity level of each patient. Then, we performed a reproducibility analysis by accounting for the duration of hospitalization (hospital days, HOD) and economic cost. In the reproducibility analysis, we measured the health cost of respiratory disease by total HOD and the total economic cost of each day and then applied the CCA. As both air pollution and the number impatient admissions were affected by the seasons, we repeated the CCA analysis between air pollution and impatient admissions after applying a regression of the mean values of each month using a dummy coding scheme (22).

#### Effect of Age and Gender

To explore the effect of age, we divided the children into two groups (below 2 years old and above 2 years old) and applied CCA to the two groups separately (23). Then, to explore the effect of gender, we divided the children into a male group and female group and applied CCA to the two groups separately.

#### Prediction Analysis

By applying the CCA method, we found that air pollution was significantly correlated with daily hospital admissions. Therefore, a support vector regression (SVR) method was applied to determine whether the data air pollution data could predict the daily hospital admissions for the upcoming day. The Libsvm 3.23 toolbox (https://www.csie.ntu.edu.tw/~cjlin/libsvm/) with default parameters was used to construct the prediction model. A leave-one-out cross validation (LOOCV) strategy was utilized to determine the performance of the prediction model. The correlation and the mean absolute error (MAE) were calculated between the original daily hospital admissions and the predicted daily hospital admissions. A permutation test with 1,000 loops was performed to assess whether the correlation coefficient was significantly higher than random (22).

$$\text{MAE}=\text{MEAN}\left(ABS\left(Inpatient_{actual}-Inpatient_{predicted}\right)\right)$$

## Results

### Description of the General Situation

A total of 10,876 inpatients with respiratory diseases were included between January 1, 2009 and December 31, 2016. The median hospitalization duration was 8 days (range from 1 to 226 days). The medium economic cost of each hospitalization was 7777.4 Yuan (range from 143.73 to 258,277.3 Yuan). The annual average SO_2_, NO_2_, PM10, and PM2.5 concentrations were 38.11, 29.30, 61.33, and 61.02 μg/m^3^, respectively (Table 1).

**Table 1:** Descriptive analysis of daily respiratory inpatient hospital admissions, the duration of hospitalization and the direct cost of hospitalization during 2009–2016.

**Variables**	**N/X ± S**	**Percentage (95%CI)**
**Number of daily respiratory inpatients (J00-J98)**	10,876	100
Acute upper respiratory infections and other		
diseases of upper respiratory tract		
(J00-J06,J30-J39)	2,347	21.58
Pneumonia and other lower respiratory diseases (J18, J20-J22, J40-J44, J46-J47)	8,300	76.31
Asthma (J45)	229	2.11
Other respiratory diseases (J60-J98)	0	0
**Age**		
0–2	8,424	77.45
2–18	2,452	22.55
**Gender**		
Male	7,037	64.7
Female	3,839	35.3
**Duration of hospitalization (days)**	9.59 ± 6.05	8 (3,20)
**Direct cost of hospitalization (RMB)**	7126.28 ± 8477.06	4711.48 (1181.91, 20020.33)
**Air pollutants**		
SO_2_ (μg/m^3^)	38.11 ± 27.57	33.56 (6, 85)
NO_2_ (μg/m^3^)	29.30 ± 14.10	27 (12, 55)
PM10 (μg/m^3^)	61.33 ± 24.86	58 (26, 104)
PM2.5 (μg/m^3^)	61.02 ± 32.81	54 (20, 125)

### Correlation Between Air Pollution and the Daily Hospital Admissions for Respiratory Disease

**Figure 1:**
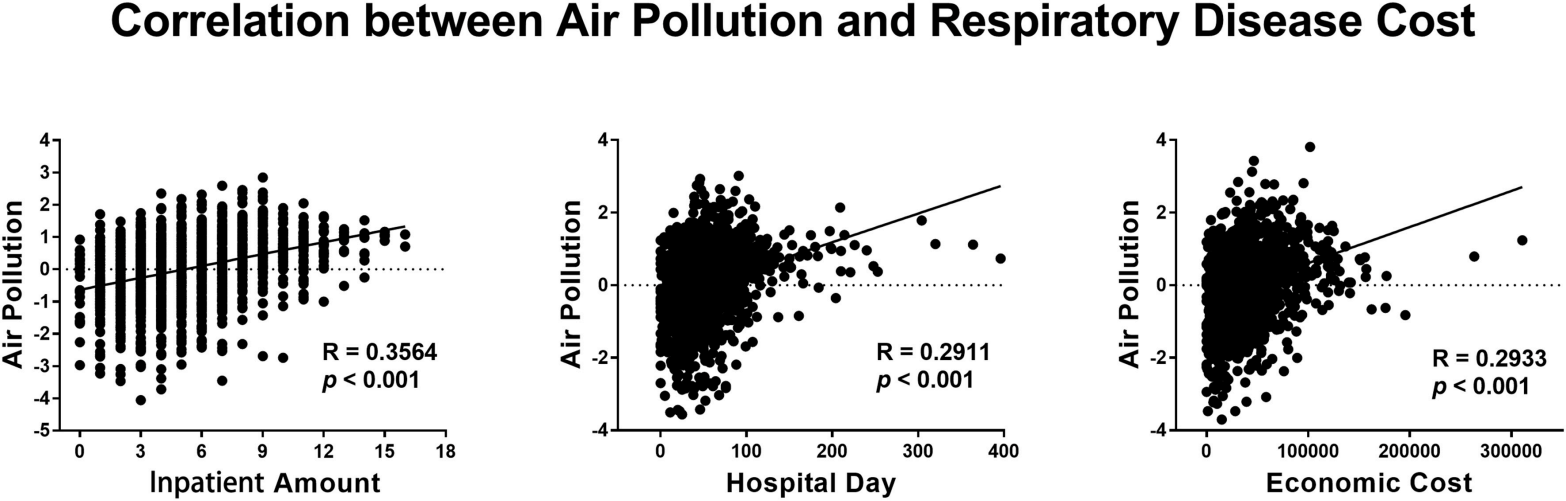
Correlation between air pollution and the health cost of respiratory disease.

Using CCA, we found significant correlations between air pollution and daily hospital admissions (*r* = 0.3564, *p* < 0.001), the duration of hospitalization (*r* = 0.2911, *p* < 0.001), and the economic cost (*r* = 0.2933, *p* < 0.001) of hospitalizations for respiratory disease (Figure 1). After regression of the mean values of SO_2_, NO_2_, PM10, PM2.5 and the number of inpatient admissions each month, we still found a significant correlation between air pollution and daily hospital admissions (*r* = 0.3734, *p* < 0.001).

### Distribution of CCA Weight

**Figure 2:**
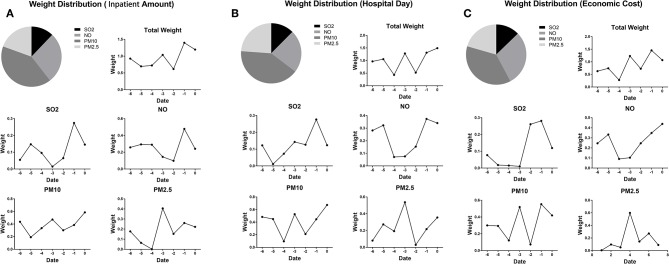
**(A)** CCA weight distribution of daily hospital admissions for respiratory diseases. **(B)** CCA weight distribution of the duration of hospitalization for respiratory diseases. **(C)** CCA weight distribution of the direct cost of respiratory diseases.

As shown in Figure 2, we found that PM10 contributed most to the inpatient cost of respiratory disease. The air pollution the day before hospitalization contributed most to the inpatient costs associated with respiratory disease. The weight distribution of each measure of air pollution is shown in Figure 2A. Single-day effects of SO_2_ and NO_2_ concentrations on daily pediatric inpatient hospital admissions reached a peak the day before hospitalization. Single-day effects of PM10 and PM2.5 concentrations reached a peak on the same day as and 3 days before hospitalization. As the daily hospital admissions might not accurately reflect the health cost, we performed a reproducibility analysis based on the duration of hospitalization and the economic cost. No major difference was found between the CCA weight distribution based on outpatient cost and the CCA weight based on the duration of hospitalization or economic cost (Figures 2B,C).

### Age Effect on the Correlation Between Air Pollution and Respiratory Disease

**Figure 3:**
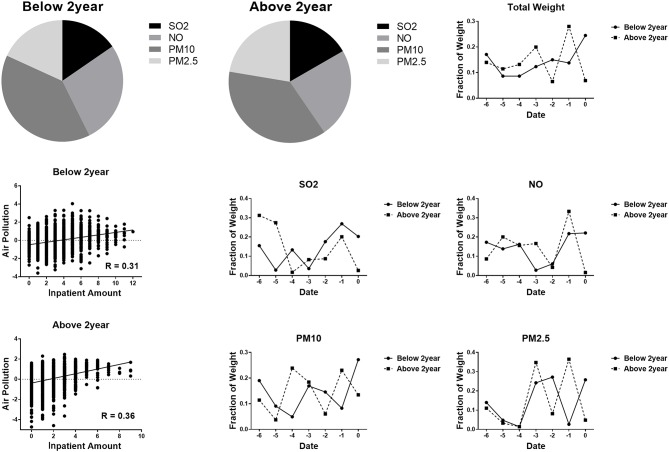
CCA weighted distribution of different ages (below 2 years and above 2 years).

To explore the age effect on the correlation between air pollution and respiratory disease, we divided the patients into two groups (below 2 years and above 2 years). There was a slightly stronger correlation between air pollution and respiratory disease in children aged 2–18 (*R* = 0.36 vs. *R* = 0.31 for children under 2 years old). The results showed a different CCA weight distribution for the two groups. The PM10 distribution weight in children below 2 years old was higher than that in the group above 2 years old. The SO_2_ distribution was lower in children below 2 years old (Figure 3).

### Gender Effect on the Correlation Between Air Pollution and Respiratory Disease

**Figure 4:**
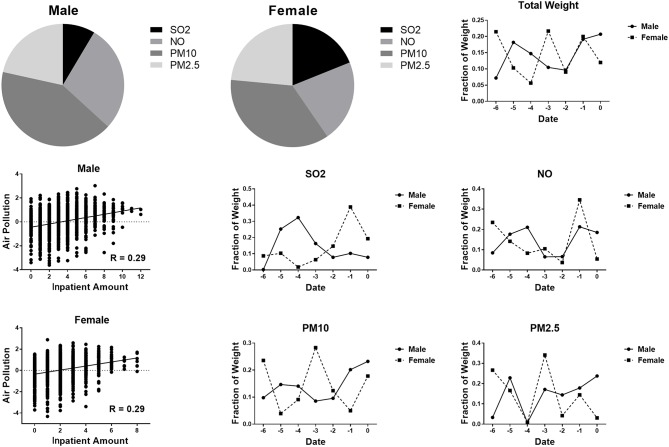
CCA weighted distribution of different genders.

To explore the gender effect on the correlation between air pollution and respiratory disease, we divided the patients into two groups (male and female patients). No significant difference was found between male and female patients. Compared to female patients, male patients were more vulnerable to PM10 concentrations and less vulnerable to SO_2_ concentrations (Figure 4).

### Prediction Analysis

**Figure 5:**
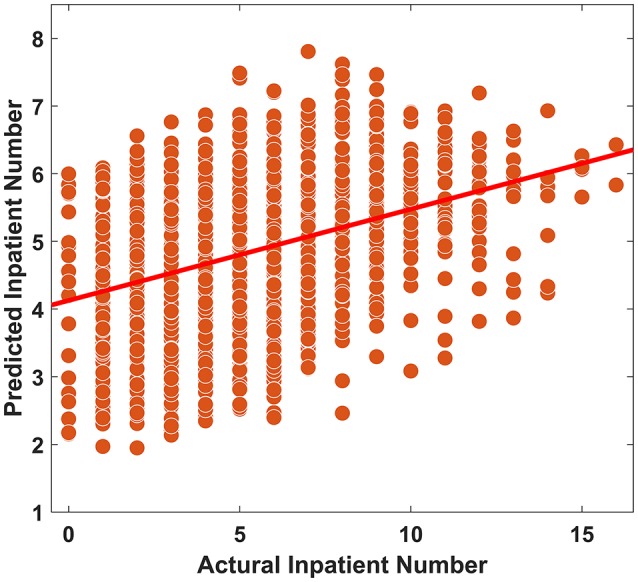
Prediction analysis between air pollution and daily pediatric inpatient hospital admissions.

As shown in Figure 5, by utilizing the SVR method, we found that air pollution could successfully predict daily pediatric inpatient hospital admissions (*R* = 0.378, permutation *p* < 0.001). The mean absolute error between the actual inpatient number and predicted inpatient number was 2.11.

## Discussion

First, this study was conducted in children in Guiyang, a less industrial city in China that was considered to be without serious air pollution. The CCA demonstrated a significant correlation between air pollution and daily pediatric inpatient hospital admissions, the duration of hospitalization and the economic cost of hospitalization for children with respiratory disease in Guiyang. Our study found that PM10 contributed most to daily pediatric inpatient hospital admissions. The air pollution the day before hospitalization contributed most to the number of respiratory disease cases. Air pollution could successfully predict daily pediatric inpatient hospital admissions.

Children are considered vulnerable to air pollutants. The longer exposure periods and greater levels of physical activity in children caused higher intake of air and deposition of particles (24). The anatomy of a developing lung and the immature immune system in children make them less able to metabolize, detoxify, and excrete toxic particles (25). Guiyang is representative of the light air pollution cities in China. However, the annual average concentrations of PM2.5, PM10, and SO_2_ were higher than the air quality guideline (AQG) recommendations by the WHO (61.02 vs. 10 μg/m^3^, 61.33 vs. 20 μg/m^3^, and 38.11 vs. 20 μg/m^3^, respectively), while the concentration of NO_2_ (29.30 μg/m^3^) reached the AQG standards (26). Compared to the previous studies conducted in heavy industrial cities such as Jinan and Lanzhou, the average air pollutant concentration in Guiyang was relatively low (8, 18). Since Guiyang is building an ecological and tourism-oriented city, the reduction in power sector emissions, industrial boiler emissions, industrial process source emissions, and transportation source emissions contribute to the relatively low concentration of NO_2_. The observed association between air pollution and hospitalization was consistent with other studies that demonstrated an increased risk for respiratory disease admissions associated with air pollution (27). Previous studies have also reported a significant association between ambient air pollution and hospital outpatient visits for respiratory diseases (19, 28). In addition, a short-term increase in hospital admission rates for chronic obstructive pulmonary disease and respiratory infection associated with PM2.5 was also observed in adults (29, 30). Those studies indicated that short-term air pollution exposure could increase the risk of hospital admission for respiratory diseases.

For the first time, the present study observed a significant association between air pollution and hospitalization cost. By 2035, it is estimated that ~2.5 million cases of non-communicable diseases will be caused by air pollution in England, making air pollution an important public health concern (12). Although air pollution has increased the burden of chronic respiratory disease in China (20), there have been few studies evaluating the association between direct hospitalization cost and air pollution. Zhang et al. reported weak or no significant associations between air pollutants and outpatient expenditure (31). The great variation in the individual cost of outpatient visits contributes to the difficulties of investigating the burden of pediatric respiratory diseases. The close correlation between hospitalization cost and disease severity made it feasible to explore the association between air pollution and hospitalization cost.

Air pollution could contribute to the pathogenesis and exacerbation of respiratory diseases, thus increasing the duration and cost of hospitalization (32). PM2.5 and NO_X_ exposure from industrial sources have been associated with decreased lung function (33). Multiple PM exposures compromise the capacity to activate the protective Nrf2 tissue defense system and result in oxidative lung damage and a systemic inflammatory reaction (34). NO_2_ promotes allergic airway disease by inducing methacholine airway hyperresponsiveness, antigen-specific IgG1 and IgE, and inflammatory leukocyte recruitment to the airway (35, 36). SO_2_ inhalation could lead to an early pulmonary response involving tissue injury, acute neutrophilic lung inflammation and airway hyperresponsiveness (37).

Different from the previous study, we found that PM10 contributed most to daily pediatric inpatient hospital admissions and the duration and cost of hospitalization. However, Tao et al. reported a greater impact of PM2.5 than PM10 on human health with the respiratory hospitalization risk increasing by 0.052% and 0.604% with an increase of 10 μg/m^3^ in PM10 and PM2.5, respectively (38). PM2.5 can absorb more harmful substances in the air and enter the respiratory system faster than PM10. It was considered to be a better indicator of lower airway exposures. The effect of PM2.5 on the number of outpatient visits for respiratory diseases was largest in the 0–18 age group (39). One possible explanation is that PM2.5 was included in the PM10 concentration, and they are highly correlated. One study found an accurate relationship between short-term PM2.5 and PM10 concentrations and the subsequent occurrence of upper respiratory tract infections by using machine learning, and PM10 showed greater accuracy for the overall population when compared to PM2.5 (40). Another possible explanation is that the effect of PM2.5 and PM10 on the respiratory tract may differ by age, which influences the anatomy and function of the respiratory system.

Similar to previous studies, a certain lag effect of short-term exposure to air pollution was revealed in the present study (19). The maximum effect occurred on the day before hospitalization. The time interval of air pollutants interfering with cellular signaling pathways and impairing cell immunity may account for the lag effect. A 0–1 day lag effect of O_3_, NO_2_, SO_2_, PM10, and CO on daily hospital admissions for respiratory disease was found in Thailand (41). Phung et al. reported that the maximum effects of air pollutants (PM10, NO_2_, and SO_2_) on the risk for hospital admissions for respiratory diseases occurred on the same day of admission (42). A non-linear artificial neural network model showed a statistically significant difference between PM2.5 exposure and hospital admission by 1 lag day (43).

In addition, a slightly stronger correlation was found among children aged 2–18 compared to those aged <2 years. One possible reason is that children aged 2–18 years spend more time outside and have higher exposure. Children below 2 years were more vulnerable to PM10 than the group above 2 years. Children below 2 years have higher ventilation rates and mouth breathing, which may pull PM10 deeper into children's lungs and the immature respiratory system, making clearance of PM10 slower and more difficult (24). Nhung et al. also proposed that long-term exposure of older children further amplifies susceptibility to the acute effects of air pollution (3). In contrast to the previous study, a gender effect was not statistically significant in our study. Previous studies suggested a gender difference in respiratory disease caused by air pollution (44, 45). However, female patients showed higher susceptibility to SO_2_ in the present study. Females were also reported to have stronger referential associations with NO_2_ and SO_2_ and hospital admissions than males (46). One potential reason is that female patients have slightly stronger airway reactivity than males (47). Moreover, the estimated respiratory disease mortality caused by air pollution was higher in females than in males (48). We also found that males were more vulnerable to PM10 than females. Males who spend more time outside and have higher ventilation rates may pull PM10 deeper into lungs (49).

### Strengths and Limitations

Our study has three strengths. First, this study analyzed air pollution and respiratory disease in a tourism-oriented city without heavy air pollution in China. Second, we analyzed the association between air pollution and hospitalization, the duration of hospitalization and the economic cost of hospitalization for children with respiratory diseases. Third, we used data for air pollutants including PM (PM2.5 and PM10), NO_2_, and SO_2_ in the present study.

However, there are several limitations in the present study. First, our study was a single-center study, and the study population was limited to children who required hospitalization, which could influence the representativeness of the study. Second, the use of average air pollution in Guiyang may have caused an exposure measurement error. Third, although the CCA showed a significant association between air pollution and hospitalization for respiratory diseases in children, the exact risk for air pollution was not calculated in the present study. Fourth, the association between each type of respiratory disease and air pollution was not analyzed in our study. Confounding factors such as the presence of respiratory viruses in children with respiratory disease were not available, which could affect the outcome. For the prediction analysis, the prediction performance was not very good and may not have achieved a level high enough for clinical use (the explanation rate = 14.19%). However, our results showed that the performance was significantly above random (permutation *p* <0.001), which suggested the potential ability to predict hospitalizations. Additionally, hospitalization is affected by multiple factors and by complex mechanisms. Future prediction studies should consider the interaction of multiple factors. Our results provide evidence that air pollution should be considered for future hospitalization prediction studies.

## Conclusions

In conclusion, air pollution was significantly associated with hospital admissions and the duration and economic cost of hospitalization in children with respiratory diseases. The maximum effect occurred on the day before hospitalization. The effect of PM10 on daily pediatric inpatient hospital admissions for respiratory disease in children was the largest among the pollutants evaluated. Air pollution could success fully predict daily pediatric inpatient hospital admissions.

## Data Availability Statement

The datasets generated for the current study were presented within the manuscript.

## Ethics Statement

The studies involving human participants were reviewed and approved by the institutional review board of Guizhou Provincial People's Hospital. Written informed consent from the participants' legal guardian/next of kin was not required to participate in this study in accordance with the national legislation and the institutional requirements. Written informed consent was not obtained from the minor(s)' legal guardian/next of kin for the publication of any potentially identifiable images or data included in this article.

## Author Contributions

HZ and YC designed the study. HZ and TW wrote the manuscript. HZ, FZ, YL, WZ, and XW collected the data. HC was the statistician on this study and revised the manuscript.

### Conflict of Interest

The authors declare that the research was conducted in the absence of any commercial or financial relationships that could be construed as a potential conflict of interest.
